# Localization optoacoustic tomography

**DOI:** 10.1038/lsa.2018.4

**Published:** 2018-04-20

**Authors:** X Luís Dean-Ben, Daniel Razansky

**Affiliations:** 1 Institute for Biological and Medical Imaging, Helmholtz Center Munich, Neuherberg 85764, Germany; 2 School of Medicine and School of Bioengineering, Technical University of Munich, Munich 81675, Germany

**Keywords:** acoustic diffraction, limited-view effects, localization, optoacoustic imaging, photoacoustic imaging, super-resolution

## Abstract

Localization-based imaging has revolutionized fluorescence optical microscopy and has also enabled unprecedented ultrasound images of microvascular structures in deep tissues. Herein, we introduce a new concept of localization optoacoustic tomography (LOT) that employs rapid sequential acquisition of three-dimensional optoacoustic images from flowing absorbing particles. We show that the new method enables breaking through the spatial resolution barrier of acoustic diffraction while further enhancing the visibility of structures under limited-view tomographic conditions. Given the intrinsic sensitivity of optoacoustics to multiple hemodynamic and oxygenation parameters, LOT may enable a new level of performance in studying functional and anatomical alterations of microcirculation.

## Introduction

Diffraction causes blurring of image features and has been traditionally associated with the spatial resolution limit in light microscopy and other imaging modalities^1^. Image resolution is defined as the smallest distance between two points that can be differentiated unambiguously. The resolution of an imaging system is quantified via its point spread function (PSF), corresponding to an image acquired from a point source. It is generally assumed that only points separated by a distance larger than the full width at half maximum (FWHM) of the PSF can be resolved. The FWHM of the PSF may erroneously be interpreted as an ‘accuracy limit’ affecting any dimensional measurement in the images. However, the precision in determining the position of an isolated source can greatly surpass the diffraction limit. Then, if individual sources can be isolated in a certain way, an image can be built by superimposing their estimated positions^2^. This idea has been successfully exploited in super-resolution fluorescence microscopy methods based on single-molecule localization, independently termed as stochastic optical reconstruction microscopy (STORM)^3^, photo-activated localization microscopy (PALM)^4^ or fluorescent photo-activation localization microscopy (FPALM)^5^. Localization microscopy has further been extended to three-dimensional imaging^6^ and recent developments have enabled a never-seen-before resolution in a few nanometers range^7^. Localization has also been used for overcoming the acoustic diffraction barrier in ultrasound (US) imaging. In this case, super-resolution is achieved by determining the position of individual microbubbles or nanodroplets^8, 9, 10^. Since such particles are moving with the blood flow, they can be localized in different positions in a sequence of B-mode images. An image of vascular structures can then be rendered by superimposing all localized sources. Ultrafast US localization has provided unprecedented images of cerebral microvessels in rodents and has further enabled the characterization of microvascular flow^11^.

Being based on light excitation and ultrasound detection, the resolution of optoacoustic (OA) imaging is affected by both optical and acoustic diffraction, typically scaling with 1/200 of the imaging depth^12^. Optical-resolution OA images can be acquired by focusing the excitation light up to a depth of ~1 mm within scattering biological tissues, where super-resolution OA methods similar to those used in fluorescence microscopy have been employed to overcome optical diffraction^13, 14, 15, 16^. At deeper regions, OA tomography (OAT) relies on acoustic inversion methods with acoustic diffraction representing an actual resolution barrier. State-of-the art OA systems based on parallel acquisition of signals for each laser pulse with ultrasound arrays have enabled three-dimensional imaging at unprecedented volumetric rates^17^. The resolution of such systems has been enhanced via processing a sequence of images acquired by slightly shifting the detection array^18^. Also, dynamic imaging of flowing individual absorbers has been showcased^19^, which was also exploited for super-resolution imaging^20^. Herein, we introduce localization optoacoustic tomography (LOT) aimed at overcoming the spatial resolution barrier of acoustic diffraction in OA imaging and tomography.

## Materials and methods

### Three dimensional optoacoustic imaging system

**Figure 1:**
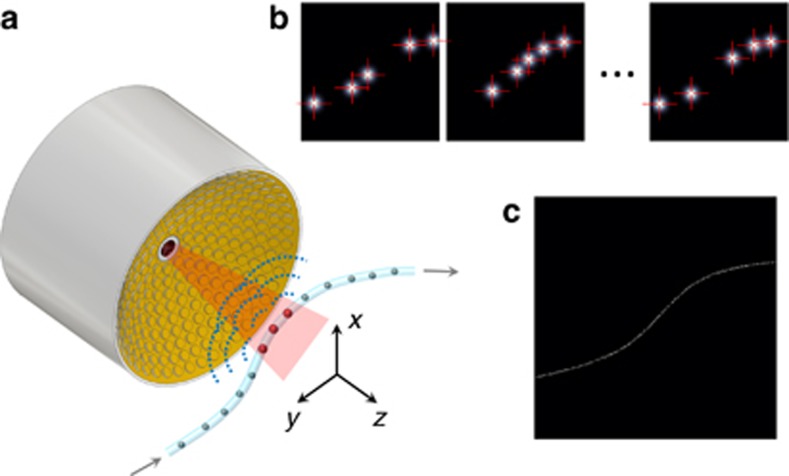
Imaging principle of localization optoacoustic tomography. (**a**) A spherical array of ultrasound transducers is used to acquire a three-dimensional optoacoustic image of flowing absorbers for each laser pulse. (**b**) The positions of sparsely distributed absorbers are measured (localized) in a sequence of images. (**c**) An image is formed by superimposing the localized positions.

LOT can in principle be performed with any OAT system providing real-time imaging capability. In order to demonstrate true 3D localization capacity, our previously reported real-time volumetric OAT system was used in the current work ^21^. It employs a spherical matrix detection array uniformly populated by 256 individual piezocomposite elements having 4 MHz central frequency and >100% bandwidth in receive mode (Figure 1). The spherical aperture has a radius of curvature of 40 mm and covers an angle of 90°. The array was fixed pointing upwards and the volume between its active detection aperture and the imaged sample was filled with agar, which served as an acoustic coupling medium and was further used to hold the imaged samples. Short-duration (<10 ns) pulses at 720-nm wavelength, generated by a tunable (700–900 nm) optical parametric oscillator (OPO) laser (Innolas Laser GmbH, Krailling, Germany), were used for the excitation of OA responses. The laser can operate at a pulse repetition frequency (PRF) of up to 100 Hz. The matrix array has a central opening through which the light beam was guided using a custom-made fiber bundle (Ceramoptec GmbH, Bonn, Germany). The detected 256 time-resolved OA signals were simultaneously digitized at 40 megasamples per second with a custom-made data acquisition system (DAQ, Falkenstein Mikrosysteme, GmbH, Taufkirchen, Germany) and transmitted to a PC via Ethernet for further processing. Synchronization of the acquisition was performed by triggering the DAQ with the Q-switch output of the laser.

### Signal and image processing

The acquired raw OA signals were first band-pass filtered for noise reduction and deconvolved with the impulse response of the transducer elements. Specifically, a Wiener filter with noise-to-signal ratio of 0 was used for the deconvolution, where the impulse response was measured by recording a signal generated by a 40 μm diameter absorbing microsphere located at the center of the spherical array ^22^. Subsequently, a second-order band-pass Butterworth filter with cut-off frequencies of 0.1 and 8 MHz was applied to the digitized signals in order to remove low-frequency bias and high-frequency noise. OA image reconstruction was then performed with a previously reported three-dimensional model-based algorithm implemented on a graphics processing unit (GPU)^23^ on a Cartesian grid of 320 × 320 × 160 voxels (16 × 16 × 8 mm^3^ FOV). In short, the OA reconstruction method consists in a numerical discretization of the OA forward model, where the pressure signals at a set of points and instants can be expressed in a vector form as


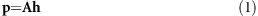


where **A** is the OA model-matrix and **h** represents the initial OA pressure at the grid of points to be reconstructed. The reconstruction algorithm is based on estimating the distribution of initial OA pressure 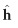 for which the measured signals **p**_m_ better match the theoretical model. This is achieved by calculating the solution of a least square problem defined as


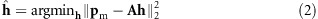


No regularization term was included in Equation (2), which was iteratively solved (10 iterations) with the LSQR method. Specific details on the numerical implementation of the reconstruction method can be found elsewhere^23^. The imaging system provides an effective field of view (FOV) of ~1 cm^3^, estimated as the volume in which the reconstructed amplitude of an absorbing particle decays by less than 50% with respect to its amplitude when located in the center of the spherical array geometry. When using the full detection bandwidth of 4 MHz of the array transducer, the tomographic system can attain spatial resolution in the 300–800 μm range in the axial (*z*) dimension and 200-700 μm in the lateral (*x*,*y*) dimensions across the 1 cm^3^ FOV^21^.

### Absorbing particles

The individual particles that were localized were ~30 μm diameter black paramagnetic polyethylene microspheres (Cospheric BKPMS 27–32). The microspheres are composed of a homogenous composition of Polyethylene and approximately 20% by weight of a Manganese Ferrous Oxide which provides paramagnetic properties as well as high light absorption for visible and near infrared wavelengths. Due to the hydrophobic nature of the spheres, they were suspended in ethanol so that they are uniformly separated in the flowing fluid.

### The optoacoustic localization method

The general concept of LOT consists of rapid acquisition of a sequence of three-dimensional OA images from flowing absorbers (Figure 1a). In this way, any structure supporting the particle flow can be accurately mapped by resolving multiple individual absorbers occupying its volume. However, absorbers smaller than the diffraction-limited resolution of the imaging system are required for resolution enhancement. If the absorbers are separated by a distance larger than the diffraction-limited resolution, their individual locations can be accurately determined in each frame of the sequence (Figure 1b). Localization of individual absorbers was performed by determining the position of local maxima in the OA images. For this, all voxels whose value is <10% of the maximum in the entire reconstructed image sequence are thresholded to 0 (~95% of all voxels). Subsequently, local maxima are determined using the built-in Matlab function imregionalmax. Note that the optimum threshold values generally depend on the particular experimental parameters, such as the laser energy, the transducer frequency or the number and type of absorbing particles. The LOT image is eventually formed by superimposing a set of points corresponding to the localized positions of the absorbers (Figure 1c).

## Results and discussion

**Figure 2:**
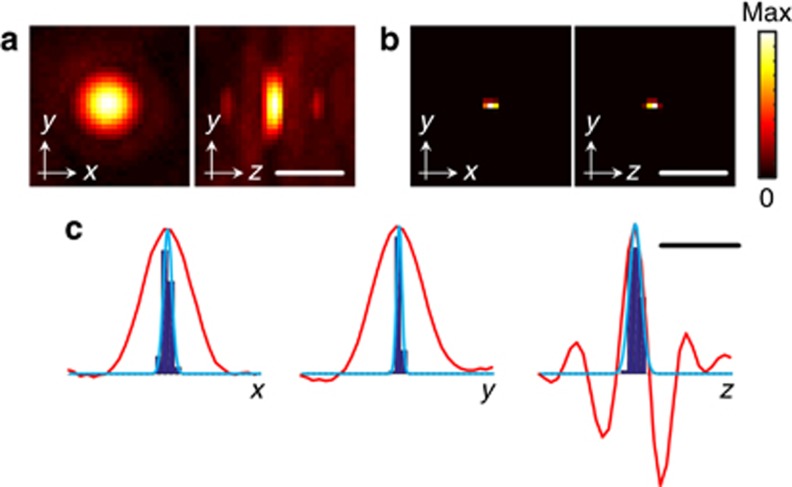
Localization accuracy. (**a**) Maximum intensity projections of the three-dimensional optoacoustic image of a 30 μm absorbing microsphere. (**b**) Equivalent image obtained as the three-dimensional histogram of the localized positions in a sequence of 5000 frames. (**c**) Normalized histograms of the localized positions in the three Cartesian coordinates (fitted Gaussian curves are shown in blue) along with the corresponding profiles (red curves) of the optoacoustic image in **a**. Scalebar 400 μm.

In a first experiment, we determined the PSF of the OAT system as well as the variations in the localized position of an individual absorber, which affects the resolution achieved with LOT. For this, a single absorbing microsphere (see Materials and methods section) was embedded in the agar matrix at approximately the center of the spherical array and a sequence of 5000 volumetric frames was acquired. The measured light fluence at the particle location was ~10 mJ cm^−2^. Due to its small size, the microsphere acts as a point absorber, thus the reconstructed volumetric image (Figure 2a) corresponds approximately to the PSF of the OAT system. Even though the PSF has a clearly defined maximum that can be taken as the localized position of the point absorber, it may not exactly match its actual position. Note that this inaccuracy does not affect the spatial resolution of LOT, which is rather determined by the ability to differentiate two adjacent point absorbers. The volumetric image built as the histogram of the localized positions of the absorber for all frames of the image sequence is displayed in Figure 2b. The histograms of the *x*, *y* and *z* coordinates of the localized positions are shown in Figure 2c along with fitted Gaussian curves. The variability in the localized position of an absorber determines the resolution that can be achieved when localizing absorbers of the same type under the same conditions. In our experiments, the standard deviation of the localized positions in the *x*, *y* and *z* directions were 78, 48 and 112 μm respectively. We ascribe the slightly higher value of the standard deviation in the *z* direction to jitter in our data acquisition system. The line profiles through the OA image along the three Cartesian coordinates are also displayed in Figure 2c. In fluorescence microscopy, the localization precision is usually reported as the standard deviation of multiple localized positions of the same source^24^. Hence, the comparison in Figure 2c illustrates the enhancement in resolution achieved with LOT as compared with standard OAT. It is also important to note that, while negative value artifacts are present in the individual OA image frames (Figure 2c, right) due to the limited-view tomographic geometry^25^, no negative values are generated in the LOT images. It can also be seen in Figure 2c that negative values in the PSF deteriorate the resolution of the OAT system in the *z* (axial) direction, which can be rectified with LOT.

**Figure 3:**
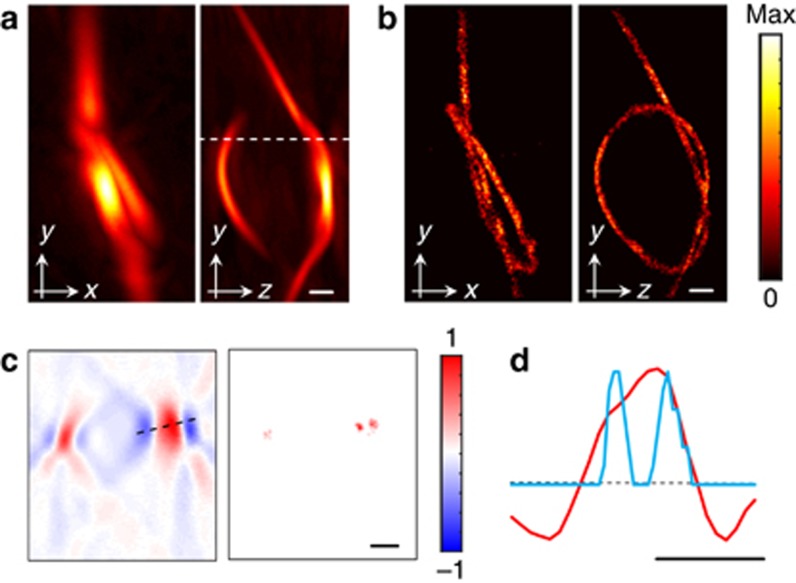
Resolution enhancement in localization optoacoustic tomography. (**a**) Maximum intensity projections of the three-dimensional optoacoustic image of a ~220 μm diameter pipette tip bent to form a knot and filled with ink. (**b**) Equivalent images obtained by localizing the positions of 3600 flowing 30 μm absorbing microspheres. (**c**) Comparison of the cross-sections marked in **a** for the standard optoacoustic image (left) and the localization optoacoustic image (right). (**d**) Comparison of the profiles marked in **c** for the standard optoacoustic image (red) and the localization optoacoustic image (blue). Scalebar 600 μm.

Localization imaging was experimentally demonstrated by suspending the 30-μm-diameter microspheres in ethanol and circulating them through a 20 μl Eppendorf microloader pipette tip (~220 μm inner diameter) bent to form a knot. A sequence of 1000 images was acquired with the laser running at a PRF of 10 Hz, which was selected to achieve the best trade-off between the average light intensity and maximum fluence, both fulfilling the safety standards for continuous human skin exposure to pulsed laser radiation^26^. At higher PRFs, the energy per pulse must be reduced in order to conform to safety limits concerning the average light intensity, resulting in a lower signal to noise ratio and hampering detection of individual absorbers. Subsequently, the pipette tip was filled with India ink (Higgins, Chartpak, optical density 20). In this case, the acquired signals were band-pass filtered between 0.1 and 3 MHz in order to emphasize the super-resolution property of the LOT method by deliberately reducing the effective diffraction-limited spatial resolution of the imaging system. The images obtained with standard OAT for the pipette tip filled with ink and those obtained with LOT are displayed in Figure 3a and 3b with their cross-sections and one-dimensional profiles shown in Figure 3c and 3d. Note that the original OA images in Figure 3 exhibit lower resolution than in Figure 2 since a narrower signal filtering bandwidth was employed (3 MHz as opposed to the full 8 MHz signal bandwidth). The LOT image was formed by considering 3600 points. Specifically, a binary image was formed by assigning a value of 1 to voxels for which at least one particle was localized to 1 and 0 otherwise. A moving average filter with kernel size 3 × 3 × 3 voxels was eventually applied to smoothen the volumetric LOT image. Rotational views of both images are provided in Supplementary Video 1. The actual formation of the super-resolution LOT image from multiple localized positions of the absorbers is further illustrated in Supplementary Video 2. It is shown that no significant differences in the LOT image are rendered for a number of points higher than ~500 (~140 frames). For a pulse repetition rate of 10 Hz, such an image can be formed in 14 seconds. Thus, in order to avoid motion artefacts in *in vivo* applications and achieve real-time LOT imaging, the acquisition rate should be significantly accelerated. Note that, while LOT clearly resolves the shape of the knot, this is not possible with the standard diffraction-limited OAT. Apart from attaining significantly better resolution beyond the diffraction limit, LOT is also responsible for the enhanced visibility of the lateral sides of the pipette tip. The lateral sides are obscured in the regular OAT images due to the limited tomographic view of the matrix detection array^25^. Thus, superimposition of multiple localized positions also aids in mitigating limited-view artefacts and eliminating negative values from the images.

In the suggested implementation, LOT is especially suited for imaging vascular structures that can support the flow of extrinsically-administered absorbers. The latter can readily be distinguished from static absorbers by considering the image differences in a sequence. Angiographic imaging is of great relevance for many applications, such as visualization of tumor neovasculature, brain function, and peripheral vascular diseases, and LOT can play an important role in the diagnosis, treatment monitoring and fundamental understanding of the mechanisms of alteration in the microcirculation. Importantly, images rendered by LOT are not affected by lack of visibility under limited-view conditions. This is often the case for hand-held OA imaging, which can only be done with a limited tomographic coverage around the imaged area. Much like other methods based on acquisition of a sequence of images of distributed absorbers^19, 27^, LOT enables visualizing vascular structures in arbitrary directions. Yet, optimal performance of LOT in living organisms is tightly linked to the development of biocompatible particles capable of generating sufficiently strong signals to be recognized in the presence of a highly-absorbing blood background. Specifically, the dynamic range of the detected OA signals must be sufficiently large to cover both the signals generated by red blood cells (RBCs) within the diffraction-limited resolution volume and the signal corresponding to the absorber to be localized. Localization can be further facilitated if the absorber can be differentiated from blood e.g. via spectral or temporal unmixing^28^. Ideally, RBCs themselves could potentially be used to achieve label-free LOT. Note, however, that RBCs occupy ~50% of the blood volume, thus their density is too high for efficient localization that generally requires relatively sparse distribution of point absorbers.

The resolution of LOT for angiographic applications is ultimately limited by the accuracy in the localized positions of individual absorbers, which could be made very high if sufficiently strong absorbers and sensitive detectors are employed to enable high SNR images with a single laser pulse excitation. In practice, the resolution is further limited by the diameter and separation between the smallest capillaries (~5 μm). While the resolution limit in localization fluorescence microscopy is generally associated with the number of photons that a molecule can emit before bleaching occurs^7^, the LOT approach exploits a much more versatile optical absorption contrast, for which highly photostable agents with long circulation time exist^28, 29^. Thus, the resolution *in vivo* is expected to be chiefly determined by the OA generation efficiency of the particular absorbing particle employed, the local light fluence and the number of localized points in the time window of interest. The same factors are also expected to affect the maximum reachable depth. The OAT imaging system employed in this work has been shown capable of imaging structures at depths exceeding 2 cm in the human breast^30^. Hence, centimeter-scale penetration is within reach for LOT as well, provided that sufficiently strongly absorbing particles are available. The digital sampling rate of the detected OA response is also of importance as it affects the maximum spatial frequency achievable in the reconstructed images. Furthermore, it is important to take into account that the PSF of the OA imaging system can be distorted due to speed of sound heterogeneities and acoustic attenuation^31, 32^, and hence further affect the best achievable resolution.

The current main limitation of LOT is the time required to form an image, which in turn depends on the frame rate and the number of points localized per image (or the corresponding particle density). It is generally not affected by the flow rate as long as it is fast enough so that absorbers are shifted in two consecutive frames. Although the temporal resolution of LOT can be optimized by increasing the absorbing particle density within the FOV, it will still remain inferior to the standard OAT where images can be acquired using single-laser shots. However, considering that OAT can readily provide a higher spatio-temporal resolution in three dimensions as compared to other bio-imaging modalities^28^, it may be still possible to image relatively fast biological events with LOT. Localization of OA signals in the time domain may also be of interest. For example, it has recently been shown that OAT can monitor neuronal activity with unprecedented spatio-temporal resolution by detecting signal variations generated by genetically encoded calcium indicators^17^. By localizing brain activation events in both the spatial and temporal dimensions, it may be possible to provide new insights into neuronal connectivity at scales and depths not accessible with fluorescence microscopy. The combined spatio-temporal information can also be used for tracking individual absorbers and measuring flow velocity when using PRFs in the order of tens of Hz^33^.

## Conclusions

The presented results demonstrate that LOT can significantly enhance the well-established advantages of OA imaging by breaking the acoustic diffraction barrier at depths within the diffusive regime of light. LOT can also attain better visibility of vascular structures, thus improve the overall image quality in limited-view tomographic acquisition scenarios. Given the intrinsic sensitivity of OA to multiple hemodynamic and oxygenation parameters, LOT may enable new level of performance in studying functional and anatomical alterations of microcirculation. OA is known for its powerful capability to attain optical absorption contrast at centimeter-scale depths with a much higher resolution than diffuse optical imaging modalities^34^. Thus, LOT can further enhance the spatial resolution of the images, enabling unprecedented capacities for deep-tissue observations, with prospective applications ranging from cancer research and dermatology to neuroscience and cardiovascular diagnostics.

## Author contributions

XL Dean-Ben and D Razansky proposed and designed the project. XL Dean-Ben performed the experiments. Both authors discussed the results and contributed to writing the manuscript.
